# Pilot Study Examining the Potential Efficacy of Music-Based Activities for People Living With Dementia in a Hospital Setting

**DOI:** 10.1192/bjo.2024.102

**Published:** 2024-08-01

**Authors:** Neha Abeywickrama, Mel N. Ellul Miraval, Hari Subramaniam, Qadeer Arshad, Elizabeta B. Mukaetova-Ladinska

**Affiliations:** 1 Leicester Medical School, University of Leicester, Leicester, United Kingdom; 2 Department of Psychology and Vision Science, University of Leicester, Leicester, United Kingdom; 3 The Evington Centre, Leicestershire Partnership NHS Trust, Leicester, United Kingdom

## Abstract

**Aims:**

Pharmacological treatment of Behavioural and Psychological Symptoms of Dementia (BPSD) is of limited benefit. The addition of non-pharmacological interventions is often essential for optimal symptom control. Music is a viable way to help patients communicate and improve quality of life. This study aims to find the most effective way to use music on a busy dementia ward.

**Methods:**

17 inpatients (aged 63–93 years) took part over a five-week period. Music with projected lyrics was individualised and based on their preferences. Instruments (e.g., maracas) were used in some group sessions. We used the Neuropsychiatric Inventory Questionnaire (NPI-Q) and Music in Dementia Assessment Scales (MiDAS) to evaluate patients’ behaviour before and after musical intervention.

**Results:**

Of NPI-Q scores, a significant difference between mean scores before and after the music intervention was found. Specifically, Delusion, Motor Disturbances, and Agitation scores were significantly reduced after music intervention. Of MiDAS, significant differences were found in Interest, Response, and Enjoyment during specific intervals.

**Conclusion:**

A multisensory inpatient environment was effective in delivering music-based activities and managed behavioural symptoms in the short term to people with advanced dementia. Its use for inpatient wards must be further investigated as an economical and personalised non-pharmacological therapeutic tool for patients with dementia.

